# Environmental Heterogeneity Leads to Spatial Differences in Genetic Diversity and Demographic Structure of *Acer caudatifolium*

**DOI:** 10.3390/plants10081646

**Published:** 2021-08-10

**Authors:** Min-Xin Luo, Hsin-Pei Lu, Min-Wei Chai, Jui-Tse Chang, Pei-Chun Liao

**Affiliations:** Department of Life Science, National Taiwan Normal University, Taipei 116059, Taiwan; xji6aup3vup@gmail.com (M.-X.L.); sp100485@gmail.com (H.-P.L.); minwei0325@gmail.com (M.-W.C.); b03612004@ntu.edu.tw (J.-T.C.)

**Keywords:** upslope shift, ecological niche modeling, genetic draft, spatial-genetic structure, paleodistribution, historical demography

## Abstract

Under climate fluctuation, species dispersal may be disturbed by terrain and local climate, resulting in uneven spatial-genetic structure. In addition, organisms at different latitudes may be differentially susceptible to climate change. Here, we tracked the seed dispersal of *Acer caudatifolium* using chloroplast DNA to explore the relationships of terrain and local climate heterogeneity with range shifts and demography in Taiwan. Our results showed that the extant populations have shifted upward and northward to the mountains since the Last Glacial Maximum. The distributional upshift of *A. caudatifolium* is in contrast to the downward expansion of its closest relative in Taiwan, *A. morrisonense*. The northern populations of *A. caudatifolium* have acquired multiple-source chlorotypes and harbor high genetic diversity. However, effective gene flow between the north and south is interrupted by topography, geographic distance, north-south differences in October rainfall, and other climate heterogeneities, blocking southward genetic rescue. In addition, winter monsoon-driven rainfall may cause regional differences in the phenological schedule, resulting in adaptive effects on the timing of range shift and the genetic draft of chlorotype distribution. Terrain, distance, and local climate also differentiate the northernmost populations from the others, supporting the previous taxonomic treatment of *Acer kawakamii* var. *taitonmontanum* as an independent variety.

## 1. Introduction

Both environmental conditions and dispersibility may be limiting factors for changes in the distribution ranges of species. The measurement of ecological niche is one way to link the suitable environmental conditions for species in which their fitness can be maintained or increased. These theoretical niche ranges are the fundamental (Grinnellian) niches [1] and could be predicted by the ecological niche modeling (ENM) under the assumption of phylogenetic niche conservatism (PNC). However, this approach does not consider life-history traits and limitations of dispersibility [2]. For sessile organisms, dispersibility could be relatively important for range change. As representative sessile organisms, plants can overcome spatial constraints through seed dispersal with variable strategies [3,4]. Consequently, seed dispersibility is one of the determinants of the ability of plants to cope with climate change.

Gene flow and demographic dynamics may be subject to spatial and environmental influences [5,6]. Given the rugged terrain distribution and steep environmental differences in mountain regions, the seed dispersal and pollen flow of mountain plants and their temporal range dynamics may jointly reflect topography and climate [7]. According to the center-periphery hypothesis [8,9], range change (e.g., spatial expansion) may be more adaptively constrained at range margins [10]. The core-to-edge decline of an adaptive genetic variation supports seed-based dispersal of mountainous maples in Taiwan rather than pollen flow [7]. However, whether the seed dispersal of maples in the same area but at lower elevations is also spatially and climate-related under climate change remains unknown.

In this study, an island maple, *Acer caudatifolium* Koidzumi, was selected as the research object. *Acer caudatifolium* is widely distributed at low to high elevations, is endemic to the island of Taiwan [11], and is a relative of temperate maples in continental Asia [12,13,14]. Taiwan is a mountainous continental island situated off the southeastern Asian Continent. Due to high seed dispersibility, the population genetic differentiation of *A. caudatifolium* is expected to be low. However, according to the mountain barrier hypothesis, mountains may hinder long-distance seed dispersal of low-elevation plants [7], resulting in population differentiation among mountain islands. In addition, the varied mountainous environment could affect population structure, interrupting gene flow. Given its massif distribution from low to high elevation and high dispersibility, *A. caudatifolium* is a suitable object for exploring the issue of spatial-environmental variation in demographic and range change.

This study asked the following two questions: (1) What are the spatial patterns of genetic diversity and population differentiation of *A. caudatifolium*? (2) Are the population genetic structure and demographic dynamics of *A. caudatifolium* associated with geographic or environmental factors? To answer these questions, we reconstructed the current and paleo distributions of *A. caudatifolium* using maternally inherited chloroplast DNA (cpDNA) to track seed dispersal and demographic dynamics, which were then correlated with geographic and environmental factors. The results illustrate how spatial and environmental heterogeneities determine the population genetic structure of mountain trees under climate change.

## 2. Materials and Methods

### 2.1. Ecological Niche Modeling

To estimate the potential distribution of *Acer caudatifolium*, we performed ecological niche modeling (ENM). We used the current distribution and projected with the same climatic variables to the middle Holocene (Holocene thermal optimal, HTO, ~6 kya) and the Last Glacial Maximum (LGM, ~21 kya) to construct the putative paleodistribution. Since the available paleoclimatic variables in the open database were limited and needed to be consistent with current variables while constructing paleo-ENM, we extracted current (1960–1990) climatic factors (19 bioclims, 24 monthly temperatures (Tmin and Tmax), and 12 monthly precipitations) in spatial resolution of 2.5 arc-minutes (approximately 4.5 km × 4.5 km at the equator) from WorldClim (https://www.worldclim.org/; accessed on 26 July 2021). The corresponding paleoclimatic variables in the HTO and LGM were also downloaded from the WorldClim database. 

Variables with variance inflation factor (VIF) >6 were removed to avoid multicollinearity using the R package usdm [15]. Only six climatic variables (bio3, bio7, prec2, prec6, prec9, and tmax2) were retained (Table S1). ENM was conducted under the maximum entropy model in MaxEnt GUI v.3.4.1 [16]. The occurrence data referred to the Global Biodiversity Information Facility (GBIF) database and our sampling records. Unreliable records (e.g., in urban regions or coasts) were discarded. To avoid extreme weighting, only one of multiple records within a grid was retained. Twenty percent of the sampling data were treated as the testing dataset. We run the ENM with 10 bootstrapped replicates by default settings (convergence threshold of 1 × 10^−5^, 10,000 background points, a maximum of 500 iterations, and a prevalence of 0.5). To evaluate the performance of the predicted niche model, we considered the area under the receiver operating characteristic curve (AUC) value from MaxEnt and also estimated the partial ROC value using NicheToolBox (http://shiny.conabio.gob.mx:3838/nichetoolb2/; accessed on 26 July 2021) with 1000 bootstrapped replicates and an E-value of 0.05.

### 2.2. Sampling and Chloroplast DNA Sequences

The population sampling included a distribution range of *A. caudatifolium* from low (381 a.s.l.) to high elevation (2987 a.s.l.), an individual has collected at least 10m away from each other without replication. For every individual, the leave was dried in silica gel and stored at 4 °C for DNA extraction. Total genomic DNA was extracted following the modified cetyltrimethylammonium bromide (CTAB) method [17] and stored at −20 °C with 1 × TE buffer. Two cpDNA fragments, *trn*H-*psb*A spacer and *rpl*16 intron, were sequenced and amplified: the former with primers 5′-GTTATGCATGAACGTAATGCTC-3′ and 5′ -CGCGCATGGTGGATTCACAATCC-3′ and the latter with primers 5′-GCTATGCTTAGTGTGTGACTCGTTG-3′ and 5′-CTTCCTCTATGTTGTTTACG-3′. The sequencing was conducted by ABIPRISMH^®^ 3730XL DNA Sequencer (Perkin-Elmer, Foster City, CA, USA) with ExoSAP-IT (Thermo Fisher Scientific Inc., Waltham, MA, USA) and the ABI BigDye 3.1 Terminator Cycle Sequencing Kit (Applied Biosystem, Foster City, CA, USA). The BioEdit [18] was applied for sequence quality check.

Concatenated chloroplast DNA (cpDNA) sequences with a total length of 1579 bp (440 bp from *trnH-psbA* and 1139 bp from *rpl16*) were generated from 294 samples in 19 populations (Table 1). Indels ≥ 2 nucleotides were recorded as a single mutation event, and 11 variant regions (sites) were ultimately generated. The obtained sequences are deposited in NCBI GenBank (accession numbers: *trnH-psbA*: MZ275974–MZ276267 *rpl16*: MZ275679–MZ275972).

### 2.3. Genetic Diversity and Haplotype Network

Indices of genetic diversity (*F_ST_*, *θs*, and *θπ*) and the site-frequency spectrum (Tajima’s *D* and Fu’s *Fs*) were estimated by Arlequin v3.5.1.3 [19]. Tajima’s *D* and Fu’s *Fs* estimated the deviance between segregating sites and nucleotide diversity to infer the demographic change against the null constant size model. The increasing rare alleles after population expansion will inflate *θs* relative to *θπ* and resulted in negative Tajima’s *D* and Fu’s *Fs*. To show the relationships between cpDNA haplotypes (chlorotypes), a pairwise-distance haplotype network was built with the minimum spanning tree (MST) algorithm in Arlequin v3.5.1.3 [19].

### 2.4. Mismatch Analysis

Mismatch analysis was performed in every polymorphic population under both demographic and spatial expansion models in Arlequin v3.5.1.3 [19]. The former model assumes population size variation (*θ*_0_ and *θ*_1_) at time *τ* with no gene flow (*m* = 0), while the latter assumes gene flow (*m*) among demes at time *τ* with constant population size (*θ*). The mismatch analysis took advantage of pairwise differences between haplotypes within populations and estimates the skewed distribution of mismatch sites to infer demography change against the null expansion model. The sum of the square deviation (SSD) and the raggedness index (Rag) was used to test the deviation from the expectation of 1000 simulations. Since the method of evaluating “expansion” in the spatial expansion model assumes *m* > 0, the “expansion” is regarded as the “range change”. In addition, because expansion times may be overestimated by orders of magnitude [20], we did not calculate the exact time of expansion *t* with the formula *t* = *τ*/2*μk* (where *μ* and *k* are the mutation rate and sequence length) but discuss the relative time (*τ*) only. 

### 2.5. Genetic Barriers

Genetic barriers were reconstructed based on geographic networks and genetic distances under Monmonier’s algorithm in the R package adegenet [21]. The method utilized Monmonier’s algorithm to identify between-group differences (i.e., barrier) based on the geographic networks and genetic distances. The K Nearest Neighbors (NN), Delaunay Triangulation (DT), and Gabriel Graph (GG) models were applied to build the population geographic connections. In the NN model, we used one-third of the data points (sampling sites) as the criterion to set the neighbors (i.e., *K* = 6). The genetic distance among populations was calculated by Nei’s distance. The optimize.monmonier function was used to compute the boundaries with 20 independent runs. The threshold of local difference was set to 0 to seek all potential barriers.

### 2.6. Genetic Differentiation across Geography and Environment

The maximum likelihood population effects mixed-effects model (MLPE) in the R package lme4 [22] was used to test whether population differentiation (gen in model) was affected by geographic distance (geo in model) (i.e., gen~geo + (1|pop), isolation by distance, IBD), adaptability to different altitudes (alt in model) (i.e., gen~alt + (1|pop), isolation by altitudinal difference, IBAlt) or different environments (env in model) (i.e., gen~env + (1|pop), isolation by environment, IBE). The combined effects among IBAlt, IBD, and IBE were also considered in model selection (Table 2). Besides the environmental variables used for ENM, we added the monthly average temperature (Tavg), solar radiation (srad), windspeed (wind), vaporization (vapr) from WorldCLim and mean global annual aridity index (GAI), actual evapotranspiration (AET), potential evapotranspiration (PET) from Global Aridity and PET Database. Among these 106 variables (Table S2), we also removing multicollinear factors with VIF values > 6 [19] and left only five factors: precipitation in October [prec10], solar radiation in June and July [srad6 and srad7], mean annual actual evapotranspiration [AET], and the global annual aridity index [GAI] (Table S1).

Genetic distance was calculated as *F*_ST_/(1 − *F*_ST_) (Table S3) [23]. The Euclidean and Canberra distances were used to calculate the geographic and altitudinal distances and the environmental differences (Tables S4–S6), respectively. The Akaike and Bayesian information criteria (AIC and BIC) values were ranked, and the model with the smallest AIC (or BIC) was selected as optimal. If two or three models had very similar AIC (or BIC) values, the likelihood ratio test was conducted to test whether the more complicated model rejected the simpler one. Subsequently, the Mantel test was performed to test whether the predictor (i.e., geographic distance) of the optimal model (i.e., IBD, see Results) significantly explained the genetic divergence. One thousand time permutations were conducted to test the significance of the correlation between the genetic and geographic distances. We also performed the partial Mantel test to exclude environmental interference when testing IBD.

### 2.7. Factors Affecting Demographic Dynamics

Population genetic patterns can also be influenced by demographic dynamics, which can be spatially and environmentally related. We, therefore, tested whether the demographic change was associated with geographic and environmental factors. The generalized linear model (GLM) was conducted in the R package stats [24], and Tajima’s *D* and the demographic or spatial expansion time (*τ*) were selected as the responses. The latitude, longitude, and altitude were taken as the geographic predictors, and the five climatic factors (prec10, srad6, srad7, AET, and GAI) were used as the environmental predictors. The optimal model was chosen using backward selection by the stepAIC function in the R package MASS [25].

## 3. Results

### 3.1. Genetic Diversity and Population Structure

**Figure 1:**
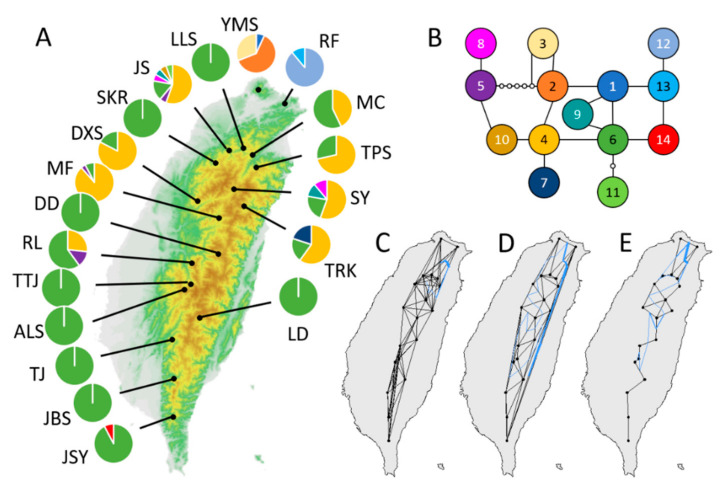
Spatial-genetic distribution of *Acer caudatifolium*. (**A**) CpDNA haplotype distribution; (**B**) haplotype network; (**C**–**E**) genetic barriers (blue lines) predicted by the Monmonier algorithm under (**C**) nearest neighbor (K = 6), (**D**) Delaunay triangulation, and (**E**) Gabriel graph. The black lines indicate the gene-flow paths allowed by the models.

The cpDNA estimation revealed that northern Taiwan is a hotspot of genetic diversity, whereas the southern populations are almost monomorphic in chlorotype (Hap6). The northern population comprises Hap4, Hap6, and their derived chlorotypes (Figure 1). Mismatch analysis showed that most of the polymorphic populations could not reject both demographic and spatial expansions according to Rag and SSD, exception the spatial expansion of the YMS and MC populations (SSD = 0.026 and 0.025, *p* = 0.001 and 0.012, respectively, Table 3 and Table S7). The extent of population expansion varied, as did the expansion times (*τ* = 0.643–8.350 and 0.135–7.924 in the demographic and spatial expansion models, respectively). These estimates do not include monomorphic populations, which does not imply constant demography of these populations but rather an inability to perform mismatch analysis.

### 3.2. Genetic Barriers

Inference of population genetic barriers by the Monmonier algorithm suggested that most of the geographic barriers are in northern Taiwan (Figure 1C–E). The NN model allowed the most corridors (connections) among populations, suggesting the fewest barriers; the DT and GG models allowed relatively fewer corridors and indicated more and similar barrier patterns. The main barrier was the same in the three models and surrounded the northeast population RF in Taiwan, although this population was still connected with the YMS population in the NN model. Both the DT and GG models isolated YMS from the other populations. RF and YMS directly face the winter northeast monsoon, which brings cold and humid air and precipitation in winter. The GG model suggested that the MF population in central Taiwan was isolated from the south. Most populations south of this barrier are monomorphic, with Hap6 as the dominant chlorotype, whereas most of the northern populations are polymorphic, with Hap4 as the dominant chlorotype (Figure 1A).

### 3.3. Geographic Distance as the Source of Population Differentiation 

Model selection in MLPE indicated that IBD had the lowest AIC and BIC, followed by IBD + IBE, which had a very similar AIC value (ΔAIC = 0.4, Table 2). IBD could not be rejected by IBD + IBE in LRT (2ΔL = 1.549, df =1, *p* = 0.213, Table S8), suggesting that IBD is the optimal (best) model explaining population genetic differentiation. Although the influences of the combined effects of geographic with environmental differences (IBD + IBE), altitudinal differences (IBD + IBAlt), and both differences (IBD + IBE + IBAlt) did not exceed geographic distance only (IBD), their AIC and logLik were close to the best IBD model (Table 2). Neither pure IBE nor IBAlt can reject IBD. However, we weighted all factors equally to explore their influence on genetic differentiation, but these factors may have different weights in reality. It must be noted that the operation of weighting factors may lead to uncertainty in the judgment of similar models. 

In analyses of Mantel and partial Mantel tests, the positive correlation between the genetic and geographic distances was only marginally significant (*p* = 0.096 and 0.059, respectively, Figure S1). Although far geographic distance does not necessarily indicate high differentiation, populations with higher genetic distances must have longer geographic distances, meaning that a long geographic distance is a necessary rather than sufficient condition for high genetic differentiation.

### 3.4. Demographic Dynamics Are Spatially and Environmentally Related

Model selection in GLM suggested that the null (empty) model could not be rejected by any alternative model in demographic expansion time. However, the optimal models for predicting the extent of population dynamics (Tajima’s *D*) and spatial expansion time comprised both spatial and environmental factors, although certain factors were excluded under backward selection (Table S9). Under these optimal models, latitude, prec10, srad6, srad7, and AET significantly predicted Tajima’s *D* (*p* = 0.003, 0.07, 0.021, 0.008, and 0.013, respectively), whereas only prec10 significantly predicted spatial expansion time (*p* = 0.010) (Table 4). Together with the Mantel test, these results indicate that although gene flow may occur between remote populations, spatial expansion is still constrained by the environment. 

### 3.5. Upward and Northward Expansion of the Distribution Range

**Figure 2:**
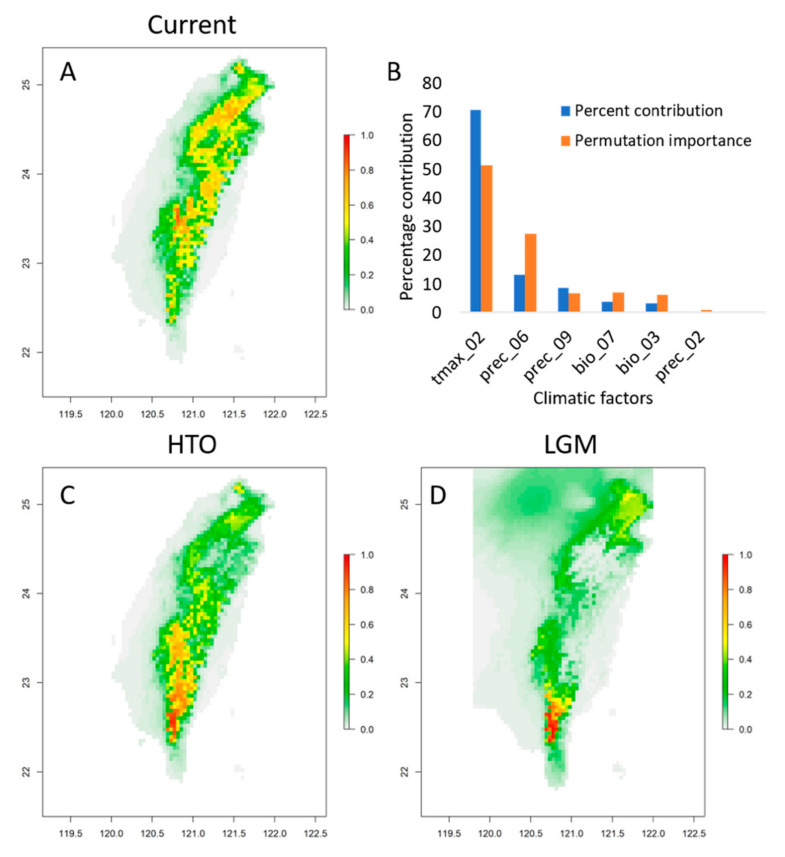
Ecological niche modeling (ENM) of the potential distribution of *Acer caudatifolium*. (**A**) Current spatial distribution; (**B**) percentage contribution of the predicted climatic factors used for current ENM; (**C**) potential distribution projected in the middle Holocene (Holocene Thermal Optimal, HTO); (**D**) potential distribution projected in the Last Glacial Maximum (LGM).

ENM predicted a potential distribution very similar to the exact distribution, with the largest percent contribution from Tmax2 (maximum temperature of February, 70.8%) (Figure 2A,B). Both AUC (mean AUC = 0.88) and partial ROC (mean = 0.913, *p* < 0.001) indicates the predicted models of *Acer caudatifolium* are reliable. The greatest contributing factor, Tmax2, was highly correlated with temperature-related Bioclims (Bio1, 6, 9, and 11), Tavg, Tmax, Tmin, vapr, AET, and PET (*r* > 0.9, *p* < 0.05) in Pearson’s correlation (Table S10). According to this map, the Yushan Mountain Range (populations TTC and ALS) is the most suitable habitat for the current distribution. When projected to the paleoclimates, the paleodistribution was restricted to the west side of the southern Central Mountain Range in the HTO, and to the southernmost Central Mountain Range in the LGM (Figure 2C,D). These predictions suggested that *A. caudatifolium* may be upward and northward to the higher-altitudinal mountainous regions with range expansion since the LGM. 

## 4. Discussion

### 4.1. Paleodistribution and Climate Change Affect the Geodistance-Related Genetic Structure

The present analysis demonstrated upward and northward expansion of the distribution of *A. caudatifolium* into higher mountain ranges from the narrow habitats in the southwestern area of Taiwan. The ENM speculates that the south was more suitable for *A. caudatifolium* growth than north-central Taiwan, although the northeast may also have potentially suitable habitats (Figure 2). Restricted range in LGM may be responsible for the spatial difference in genetic diversity between northern and southern Taiwan. The north-south separation of ancestral distribution is also reflected in the genetic differentiation related to the geographical distance between the north and south populations (IBD model). The high genetic variation and differentiation in the north and monomorphic characteristics in the south indicate a correlation of latitude with demography. In addition, the population structure and spatial-temporal expansion are affected by multiple climatic factors, revealing an environmental impact of spatial-genetic structure on island mountain trees, i.e., environmentally driven genetic draft [26,27]. Climate-related population structure has also been observed in Taiwan for skullcap flowers [28,29], *Rhododendron* [30,31], and cow-tail fir [32]. However, the gene flow of *A. caudatifolium* is still related to geographic distance, albeit marginally. However, geographic distance alone may not be sufficient to explain the population differentiation caused by genetic drift in the short term. Other factors, such as the spatial and temporal differences of local climate, may also limit population distribution and gene flow. In other words, climate change determines species distribution, and geographic constraints and environmental heterogeneity affect genetic distribution.

### 4.2. Climate Change Facilitates the Contact of Two Closely Related Maples with Divergent Grinnellian Niches

According to PNC, the distribution range of the most suitable habitat varies temporally with climate change. ENM in this study indicated that *A. caudatifolium* upward shifted to mountain ranges after the LGM (Figure 2), in contrast to the downward expansion of *A. morrisonense* from high mountains (Figure 5 of [7]). Among all maples in Taiwan, these two species have the closest phylogenetic relationship but are not sister species [12,13,14]. It can be inferred that phylogenetic niche divergence between these two species caused their respective ancestors to occupy different territories when entering Taiwan. Subsequent climate warming might facilitate their distributional contact, increasing competitive interaction, although such competition is likely to be modest [33]. However, further study is required to establish the direction of the causal relationship, that is, whether niche divergence promotes distributional contact leading to competitive pressure [33] or the competition caused by distributional contact leads to niche divergence [34]. 

Like *A. morrisonense*, the chlorotypes of *A. caudatifolium* are differentiated from north to south (Figure 2b of [7]). However, the north-south differentiation of *A. caudatifolium* resulted from the collection of different ancient populations (Figure 2), whereas divergent alpine refugia were responsible for the differentiation of *A. morrisonense* (Figure 5 of [7]). During the LGM, the west side of the southern Central Mountain Range of Taiwan were most suitable for *A. caudatifolium*, and northeastern Taiwan was another appropriate range separated from the southwest by the Central Mountain Range. The ancient northeastern populations harbored diverse chlorotypes, whereas the ancient southern populations had low genetic diversity. Under climate warming, these temperate-origin maples shifted to higher altitudes accompanied by range expansion. Therefore, the extant northern populations have chlorotypes from both the ancient southwestern and northeastern populations. By contrast, the southern populations only retain the original southwestern chlorotype due to geographic barriers. 

### 4.3. Environmentally Biased Dispersal Constrains the Northernmost Populations

The distinct chlorotype composition suggests the northernmost populations as a unique evolutionary significant unit from the southern populations. The formation of the northeast-southwest geographic barrier may be attributable to environmentally based dispersal bias. The disruption of gene flow by terrain according to the mountain-barrier effect is more significant in the northern populations, particularly for blocking gene flow between the southern and northernmost populations on and around the Datun volcano group (i.e., populations YMS and RF). Environmentally determined migratory characters, e.g., seed dispersibility, may constrain the extent of range shift. Although the seeds of *A. caudatifolium* have wings that aid spreading, maple seed dispersal is unexpectedly distance-limited and aggregated [35]. The warming-driven downslope range shift is also more limited than the upshift [36], resulting in the limited passage of mountainous maples through the lower basins and lowlands and interrupting gene flow between the Datun volcano group and the southern populations. 

Northeastern Taiwan is affected by the cold and humid winter northeast monsoon from October to April of the next year. The local climate difference (e.g., prec10 and Tmax2) between the rainy northeast and arid southwest in Taiwan has affected forest phenology [37,38]. Xylem embolism of maples under water deficiency may cause leaf senescence [39], facilitating leaf color change and defoliation. For deciduous trees, the duration of green leaves affects nutrient accumulation and reproductive yield in the following year [40,41]. The leaves of *A. caudatifolium* typically turn yellow in October, and differences in late autumn rainfall between northeastern and southwestern Taiwan may result in differences in leaf duration and next-year seed yield (i.e., fitness). The significant correlation between prec10 and the timing of range change implies that autumn rainfall was one of the key factors affecting adaptive migration under paleoclimate change. In addition, since selection may eliminate maladaptive migrants, such a phenological difference may affect the success of colonization, thereby interrupting north-south gene flow. The greatest contributing factor of ENM, Tmax2, are highly correlated with other temperature-related factors (Bio1, 6, 9, 11, Tavg1~5, 11, 12, Tmax1~5, 10~12, Tmin1~3, 12, vapr1~5, 10~12, AET, and PET, Table S10). These climate factors are mostly related to the winter temperature, highlighting the impact of the temperature in the winter northeast monsoon period on the current distribution of *A. caudatifolium* and on the maintenance of genetic differences between southern and northern populations.

### 4.4. Local Climate Heterogeneity Underlies Genetic Draft of Chlorotype Distribution

Our results show that local climate heterogeneity combined with the influence of geography (i.e., terrain and distance) reduces the ability of the northernmost chlorotypes to enter the south, resulting in genetic differentiation from the south. Prec10, srad6, srad7, and AET were related to Tajima’s *D*, indicating that autumn rainfall, summer sunshine, and evapotranspiration jointly determine the extent of population renewal and demographic structure. Precipitation, solar radiation, and evapotranspiration may affect photosynthesis, nutrient accumulation, growth, and yield simultaneously. These environmental factors related to survival and reproduction are undoubtedly crucial to population genetic structure via local adaptation and affect the chlorotype distribution via the genetic draft, particularly for populations in central to northern Taiwan. 

Seasonality is more pronounced in northern Taiwan than in southern Taiwan. The higher genetic diversity of the northern populations promotes their adaptability and enables local adaptation to long-term seasonal and climate changes. By contrast, the southern margin may be more susceptible to climate change [42], as reflected in the reduced distributions after the warming in the LGM. Warming can even reduce the competitiveness of organisms [43], leading to more severe genetic losses in the south. Therefore, in the face of global warming, the potential of the lack of genetic variation in the south to weaken resilience deserves further attention. 

## Figures and Tables

**Table 1:** Sampling site information and summary statistics of genetic diversity.

Pop	Longitude (E)	Latitude (N)	Altitude (m)	*N*	Polym	avg*F_ST_*	*Θs* *	SD(*θs*) *	*Θπ* *	SD(*θπ*) *	Tajima’s *D*	*P*	Fu’s *Fs*	*P*
YMS	121.539	25.181	762–1021	29	2	0.724	0.323	0.239	0.365	0.338	0.273	0.688	0.326	0.527
RF	121.787	25.065	381–484	9	1	0.432	0.233	0.233	0.141	0.207	−1.088	0.198	−0.263	0.190
LLS	121.401	24.687	1166–1317	7	0	0.462	0	N.A.	0	N.A.	0	N.A.	0	N.A.
JS	121.27	24.667	1197–1468	17	8	0.223	1.499	0.721	1.402	0.910	−0.229	0.461	−0.295	0.454
MC	121.486	24.628	1106–1218	21	1	0.436	0.176	0.176	0.326	0.319	1.505	0.950	1.474	0.733
SKR	121.143	24.56	1546–1975	20	0	0.300	0	N.A.	0	N.A.	0	N.A.	0	N.A.
TPS	121.521	24.523	1566–1764	14	1	0.238	0.199	0.199	0.278	0.297	0.842	0.850	0.944	0.572
SY	121.312	24.338	1848–2000	10	8	0.509	1.791	0.924	1.337	0.919	−1.094	0.176	0.713	0.637
DXS	120.977	24.236	1680–2026	23	1	0.170	0.172	0.172	0.190	0.229	0.186	0.769	0.612	0.438
TRK	121.407	24.192	2336–2987	5	2	0.338	0.608	0.480	0.507	0.505	−0.973	0.189	−0.829	0.106
MF	121.176	24.093	2087–2283	26	8	0.329	1.328	0.611	0.435	0.380	−2.115	0.002	0.610	0.586
DD	121.169	23.787	2196–2396	4	0	0.251	0	N.A.	0	N.A.	0	N.A.	0	N.A.
RL	120.922	23.708	1362–1642	15	8	0.367	1.558	0.761	1.423	0.930	−0.318	0.436	3.081	0.935
TTC	120.913	23.53	1604–2388	17	0	0.289	0	N.A.	0	N.A.	0	N.A.	0	N.A.
ALS	120.855	23.482	1930–2404	15	0	0.886	0	N.A.	0	N.A.	0	N.A.	0	N.A.
LD	120.995	23.245	2033–2309	26	0	0.339	0	N.A.	0	N.A.	0	N.A.	0	N.A.
TJ	120.741	23.06	1467–1490	2	0	0.348	0	N.A.	0	N.A.	0	N.A.	0	N.A.
JBS	120.757	22.726	1306–2040	21	0	0.264	0	N.A.	0	N.A.	0	N.A.	0	N.A.
JSY	120.75	22.4	1257	13	1	0.352	0.204	0.204	0.097	0.163	−1.149	0.169	−0.537	0.128

*N*, sample size; Polym, number of polymorphic sites (including indels). * These values have been multiplied by 1000.

**Table 2:** Summary results of model selection in MLPE. The models are listed in order of AIC value; the IBD model has the best performance.

Model	Formula	npar	AIC	BIC	logLik	Deviance
IBD	gen~geo + (1 | pop)	4	1103.3	1115.8	−547.630	1095.3
IBD + IBE	gen~geo + env + (1 | pop)	5	1103.7	1119.4	−546.860	1093.7
IBD + IBAlt	gen~geo + alt + (1 | pop)	5	1105.1	1120.8	−547.540	1095.1
IBD + IBE + IBAlt	gen~geo + env + alt + (1 | pop)	6	1105.7	1124.5	−546.850	1093.7
IBE	gen~env + (1 | pop)	4	1110.7	1123.3	−551.350	1102.7
IBAlt	gen~alt + (1 | pop)	4	1111.5	1124.0	−551.740	1103.5
IBE + IBAlt	gen~env + alt + (1 | pop)	5	1112.6	1128.3	−551.300	1102.6

npar, number of parameters; gen, genetic distance; geo, geographic distance; env, environmental difference; alt, altitudinal difference.

**Table 3:** Summary results of mismatch analysis under the demographic and spatial expansion models. Standard deviation (SD) and 95% confidence intervals (95% CI) are displayed in Table S7.

	Demographic Expansion					Spatial Expansion				
Pop	*τ*	*θ*_0_	*θ*_1_	SSD	Rag	*τ*	*θ*	*M*	SSD	Rag
YMS	0.744	0	999	0.026	0.199	0.742	0.003	999	0.026	0.199
RF	2.930	0.900	3.600	0.307	0.358	0.260	0.008	999	0.0005	0.358
JS	8.350	0	1.642	0.043	0.112	7.099	1.381	0.31	0.035	0.112
MC	0.789	0.002	999	0.025	0.265	0.785	0.006	999	0.025	0.265
TPS	0.643	0	999	0.012	0.208	0.645	0.001	999	0.012	0.208
SY	1.031	0.004	999	0.037	0.129	7.897	1.69	0.159	0.063	0.129
DXS	2.982	0.900	3.600	0.238	0.250	0.387	0.003	999	0.002	0.250
TRK	1.037	0	999	0.065	0.350	1.035	0.003	999	0.065	0.350
MF	3	0	0.247	0.007	0.439	7.646	0.208	0.079	0.005	0.439
RL	0.725	0.010	999	0.058	0.166	7.924	1.009	0.326	0.057	0.166
JSY	2.965	0.450	0.450	0.028	0.503	0.135	0.110	2.705	0.0002	0.503

LLS, SKR, DD, TTC, ALS, LD, TJ, and JBS are genetically monomorphic, and thus mismatch analysis could not be conducted.

**Table 4:** Summary results of the generalized linear model for testing significant factors in the extent of demographic dynamics (Tajima’s *D*) and spatial expansion time (*τ*).

	Tajima’s *D*				Spatial Expansion Time (*τ*)			
	Estimate	SE	*t*	*P*	Estimate	SE	*t*	*P*
Lat	2.856	0.741	3.853	0.003 *	−6.626	3.613	−1.834	0.090
Long	−2.681	1.697	−1.580	0.143	17.710	8.358	2.119	0.054
Alt	NA	NA	NA	NA	−0.006	0.003	−1.955	0.072
prec10	0.013	0.004	3.279	0.007 *	−0.051	0.017	−3.025	0.010 *
srad6	0.005	0.002	2.691	0.021 *	NA	NA	NA	NA
srad7	−0.004	0.001	−3.220	0.008 *	NA	NA	NA	NA
AET	−0.015	0.005	−2.972	0.013 *	−0.035	0.028	−1.250	0.233
GAI	−0.0001	0.00004	−1.775	0.104	NA	NA	NA	NA

* *p* < 0.05.

## Data Availability

The cpDNA sequences are available on NCBI GenBank (accession numbers: *trnH-psbA*: MZ275974–MZ276267 *rpl16*: MZ275679–MZ275972).

## Supplementary Materials

The following are available online at https://www.mdpi.com/article/10.3390/plants10081646/s1, Table S1: Variance inflation factors (VIFs) of the retained environmental variables; Table S2: Environmental factors of the 19 populations of *Acer caudatifolium* used in this study (csv file); Table S3: Population Pairwise genetic distances *F_ST_*/(1-*F_ST_*) matrix (csv file); Table S4: Population pairwise geographic distances matrix (csv file); Table S5: Population pairwise altitudinal distances matrix (csv file); Table S6: Population pairwise environmental differences matrix (csv file); Table S7: Details of the estimated indices of mismatch analysis; Table S8: Likelihood ratio test comparing the best two models in MLPE; Table S9: The optimal model used for generalized linear model (GLM) testing of the factors affecting demography; Table S10: Pearson’s correlation of environmental factors (csv file); Figure S1: Correlations between geographic distance and genetic differentiation according to the Mantel test and partial Mantel test conditioned on environmental differences.
