# Supplementary material for: Environmental Heterogeneity Leads to Spatial Differences in Genetic Diversity and Demographic Structure of Acer caudatifolium
Source: Plants (Basel). 2021 Aug 10;10(8):1646. doi: 10.3390/plants10081646 (PMC8398000; doi:10.3390/plants10081646)

Figure S1. Correlations between geographic distance and genetic differentiation according to the Mantel test and partial Mantel test conditioned on environmental differences.

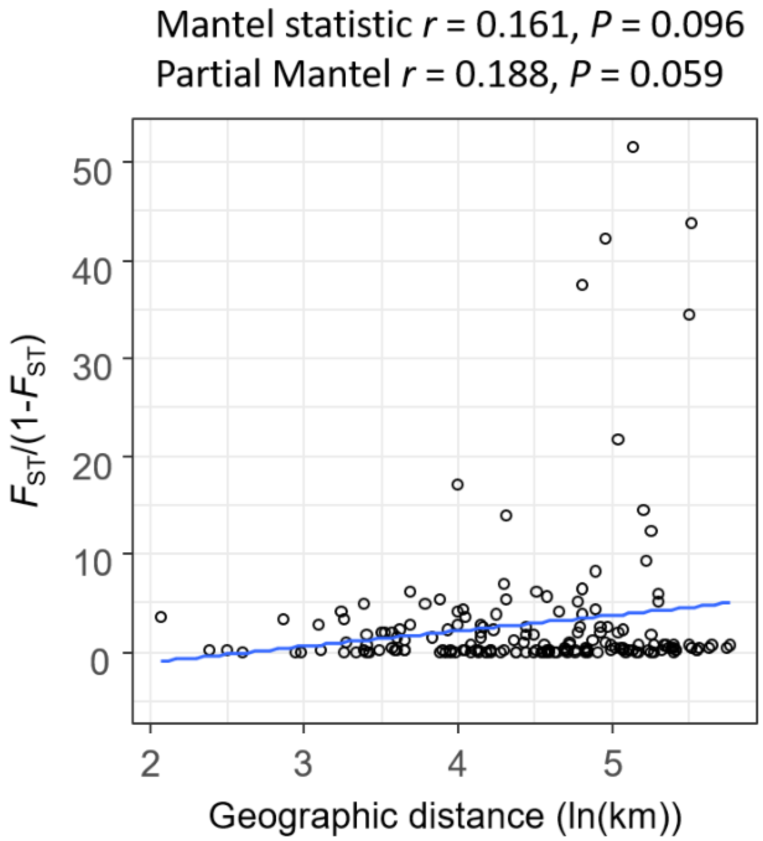

Supplement: Supplementary file 1 [file plants-10-01646-s001.zip › Figure S1.pdf]
