# Supplementary material for: Environmental Heterogeneity Leads to Spatial Differences in Genetic Diversity and Demographic Structure of Acer caudatifolium
Source: Plants (Basel). 2021 Aug 10;10(8):1646. doi: 10.3390/plants10081646 (PMC8398000; doi:10.3390/plants10081646)
Supplement: Supplementary file 1 [file plants-10-01646-s001.zip › Table S1.pdf]

Table S1. Variance inflation factors (VIFs) of the retained environmental variables.

Variables	VIF
Retained variables for ENM	
bio 03	2.540445
bio 07	4.385546
prec 02	4.792062
prec 06	1.96393
prec 09	1.612133
tmax 02	3.158953
Retained variables for MLPE and GLM	
prec 10	3.204929
srad 6	2.447941
srad 7	2.472712
AET	3.370432
GAI	2.043546
