# Supplementary material for: Environmental Heterogeneity Leads to Spatial Differences in Genetic Diversity and Demographic Structure of Acer caudatifolium
Source: Plants (Basel). 2021 Aug 10;10(8):1646. doi: 10.3390/plants10081646 (PMC8398000; doi:10.3390/plants10081646)
Supplement: Supplementary file 1 [file plants-10-01646-s001.zip › Table S7.pdf]

Table S7. Details of the estimated indices of mismatch analysis.

	Demographic expansion										Spatial expansion									
Pop	τ	95%CI	θ_0	95%CI	θ_1	95%CI	SSD	P	Rag	P	τ	95%CI	θ	95%CI	M	95%CI	SSD	P	Rag	P
YMS	0.744	0.244-1.299	0	0-0.026	999	7.906-999	0.026	0.077	0.199	0.073	0.742	0.374-1.455	0.003	0.001-0.466	999	1.068-999	0.026	0.001	0.199	0.087
RF	2.930	0-87.930	0.900	0-3.600	3.600	2.342-999	0.307	0.101	0.358	0.258	0.260	0.00001-1.497	0.008	0.001-0.051	999	0.00001-999	0.0005	0.485	0.358	0.693
LLS	0	-	0	-	0	-	0	0	0	0	0	-	0	-	0	-	0	0	0	0
JS	8.350	0.639-91.350	0	0-0.981	1.642	0.274-999	0.043	0.557	0.112	0.680	7.099	0.095-59.529	1.381	0.001-2.711	0.310	0.139-999	0.035	0.380	0.112	0.773
MC	0.789	0.102-1.395	0.002	0-0.060	999	13.863-999	0.025	0.087	0.265	0.062	0.785	0.333-1.828	0.006	0.001-0.729	999	0.334-999	0.025	0.012	0.265	0.070
SKR	0	-	0	-	0	-	0	0	0	0	0	-	0	-	0	-	0	0	0	0
TPS	0.643	0-1.322	0	0-0.035	999	14.117-999	0.012	0.335	0.208	0.297	0.645	0.180-48.290	0.001	0.001-0.637	999	0.157-999	0.012	0.170	0.208	0.297
SY	1.031	0.205-1.953	0.004	0-0.028	999	6.607-999	0.037	0.192	0.129	0.573	7.897	0.110-75.600	1.690	0.001-3.024	0.159	0.199-999	0.063	0.235	0.129	0.742
DXS	2.982	0-87.982	0.900	0-3.714	3.6	2.315-999	0.238	0.131	0.250	0.302	0.387	0.123-1.294	0.003	0.001-0.309	999	0.191-999	0.002	0.224	0.250	0.429
TRK	1.037	0-2.398	0	0-0	999	5.711-999	0.065	0.251	0.350	0.516	1.035	0.00001-2.616	0.003	0.001-0.630	999	0.00001-999	0.065	0.257	0.350	0.504
MF	3.000	0.408-3.5	0	0-0	0.247	0-999	0.007	0.314	0.439	0.616	7.646	0.00001-50.531	0.208	0.001-0.166	0.079	0.00001-999	0.005	0.372	0.439	0.672
DD	0	-	0	-	0	-	0	0	0	0	0	-	0	-	0	-	0	0	0	0
RL	0.725	0-1.457	0.010	0-0.049	999	6.666-999	0.058	0.104	0.166	0.366	7.924	0.00001-85.820	1.009	0.001-2.695	0.326	0.00001-999	0.057	0.239	0.166	0.703
TTC	0	-	0	-	0	-	0	0	0	0	0	-	0	-	0	-	0	0	0	0
ALS	0	-	0	-	0	-	0	0	0	0	0	-	0	-	0	-	0	0	0	0
LD	0	-	0	-	0	-	0	0	0	0	0	-	0	-	0	-	0	0	0	0
TJ	0	-	0	-	0	-	0	0	0	0	0	-	0	-	0	-	0	0	0	0
JBS	0	-	0	-	0	-	0	0	0	0	0	-	0	-	0	-	0	0	0	0
JSY	2.965	0.359-13.465	0.450	0-0.554	0.450	0-999	0.028	0.170	0.503	0.429	0.135	0.00001-0.969	0.110	0.001-0.110	2.705	0.00001-999	0.0002	0.406	0.503	0.697

Mismatch analysis of LLS, SKR, DD, TTC, ALS, LD, TJ, and JBS could not be performed due to monomorphism.
