# Supplementary material for: Environmental Heterogeneity Leads to Spatial Differences in Genetic Diversity and Demographic Structure of Acer caudatifolium
Source: Plants (Basel). 2021 Aug 10;10(8):1646. doi: 10.3390/plants10081646 (PMC8398000; doi:10.3390/plants10081646)
Supplement: Supplementary file 1 [file plants-10-01646-s001.zip › Table S8.pdf]

Table S8. Likelihood ratio test comparing the best two models in MLPE.

Model	npar	logLik	χ^2 (2ΔL)	df	*P*
IBD	4	-547.63			
IBD+IBE	5	-546.86	1.5489	1	0.2133

npar, number of parameters.
