# Supplementary material for: Environmental Heterogeneity Leads to Spatial Differences in Genetic Diversity and Demographic Structure of Acer caudatifolium
Source: Plants (Basel). 2021 Aug 10;10(8):1646. doi: 10.3390/plants10081646 (PMC8398000; doi:10.3390/plants10081646)
Supplement: Supplementary file 1 [file plants-10-01646-s001.zip › Table S9.pdf]

Table S9 The optimal model used for generalized linear model (GLM) testing of the factors affecting demography

Response	Predictors
Tajima's *D*	~ Lat + Long + prec_10 + srad_06 + srad_07 + AET + GAI
τ (demographic expansion time)	~ 1 (null model)
τ (spatial expansion time)	~ Lat + Long + Alt + prec_10 + AET
